# A systematic review of analgesia practices in dogs undergoing ovariohysterectomy

**DOI:** 10.14202/vetworld.2018.1725-1735

**Published:** 2018-12-25

**Authors:** Willy E. Mwangi, Eddy M. Mogoa, James N. Mwangi, Paul G. Mbuthia, Susan W. Mbugua

**Affiliations:** 1 Department of Clinical Studies, Faculty of Veterinary Medicine, University of Nairobi, Nairobi, Kenya; 2 Department of Veterinary Pathology, Microbiology, and Parasitology, Faculty of Veterinary Medicine, University of Nairobi, Nairobi, Kenya

**Keywords:** analgesia practices, dogs, outcomes, ovariohysterectomy, systematic review

## Abstract

**Aim:**

This was a systematic review conducted to evaluate the analgesic drugs and techniques used in the management of pain in dogs undergoing ovariohysterectomy.

**Materials and Methods:**

Systematic searches in PubMed, Google Scholar, and ScienceDirect were conducted for peer-reviewed articles written in English and published from 1995 to 2015. The key search words were dogs, ovariohysterectomy, pain, and analgesics. This was followed by a manual search of the references within the primary data sources. Inclusion and exclusion of studies and data extraction were performed independently by two reviewers. All randomized studies evaluating the effects of analgesics during ovariohysterectomy in dogs were included.

**Results:**

A total of 31 trials met the criteria and were, therefore, included in the study. Data on the type of analgesic drugs used, the technique of administration, and the need for rescue analgesia were extracted from the papers. Individual analgesic protocols were used in 83.9% of the studies compared to multimodal drug therapy, which was used in 16.1% of the studies. Opioids were used in 39.0% of studies, nonsteroidal anti-inflammatory drugs (NSAIDs) in 19.4%, a combination of NSAIDs and opioids in 19.4%, local analgesics in 6.5%, and acupuncture in 3.2% of the studies. Drug administration was done using three approaches that included pre-operative (64.5%), post-operative (22.6%) as well as combined pre- and post-operative approach (12.9%). In 77.4% of the studies, administration of analgesics was done once, while in 12.9%, it was done as a 72-h post-operative course. 24-h and 48-h courses of post-operative pain therapy were done in 6.5% and 3.2% of the studies, respectively. About 57% of the dogs in the control groups required rescue analgesia as compared to 21.6% in the single and 11.3% in multimodal drug therapy groups. The requirement for rescue analgesics was highest in dogs treated using acupuncture (43.8%) and lowest in dogs treated using NSAID-opioid combination (8.6%). Fewer dogs among those that received pain medication preoperatively and postoperatively required rescue analgesia compared to those in groups given drugs before and after surgery only. More dogs (26.4%) among those given analgesics only once postoperatively required rescue analgesia as compared to those that received analgesics daily for 72 h (4.4%).

**Conclusions:**

This study provides evidence that opioids are the mainstream analgesic drugs used in managing acute post-operative pain in dogs’ post-ovariohysterectomy. In addition, multimodal drug therapy, particularly, NSAID-opioids combination is more effective for pain management than single drug administration. Administering analgesics both before and after surgery is associated with better outcomes and so is a protracted course of post-operative pain therapy. Although these practices should be encouraged, controlled studies should be conducted to conclusively determine the best practices for pain management in dogs undergoing ovariohysterectomy.

## Introduction

Ovariohysterectomy is a routine surgical procedure which is known to cause marked acute pain in dogs [1]. Perioperative analgesia in surgical patients is paramount not only for humane and ethical considerations but also for the reason that it helps to minimize the deleterious physiological effects associated with pain [2]. These harmful effects include increased post-operative stress, immunosuppression, increased arterial blood pressure, delayed wound healing, negative protein balance, decreased food intake, and development of maladaptive behaviors including self-mutilation [3,4].

The numerous analgesic drugs and techniques currently available for the management of pain in animals pose a challenge to practicing clinicians with regard to the choice of the appropriate drug and technique for optimal pain management in animals. Practically, the choices are mainly influenced by the type of surgery, past experiences of the clinicians and their knowledge of the specific drug or technique, availability of the drug, associated side effects, cost, and occasionally set guidelines for the clinic or hospital [5].

This study evaluated the trends in analgesia practices in dogs undergoing ovariohysterectomy and further determined their effectiveness in managing post-operative pain. The results of this systematic review can help the clinician to make an informed decision on the most appropriate choice of analgesics and techniques for effective pain management, hence leading to better animal welfare and favorable surgical outcomes.

## Materials and Methods

### Ethical approval

This is a systematic review, hence no ethical approval is necessary.

### Data search

A literature search was conducted to identify all trials comparing or testing the efficacy of analgesics used in managing post-operative pain in dogs after ovariohysterectomy. Systematic searches in three databases, namely PubMed, Google Scholar, and ScienceDirect, were conducted for peer-reviewed articles written in English and published between 1995 and 2015. The literature search was designed to retrieve all articles using dogs, ovariohysterectomy, pain, and analgesics as the key search words. This was followed by a manual search of the references within the primary data sources to get more articles that might not have been picked using the three databases.

### Inclusion and exclusion of studies

**Figure-1:**
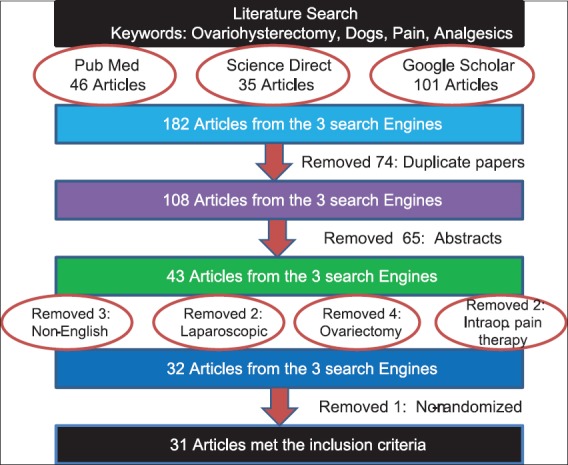
The systematic criteria used to exclude and include the articles in this study.

All studies published from 1995 to 2015, written in English and assessing the effectiveness of analgesics in managing pain after ovariohysterectomy in dogs, were included. Studies with controlled or uncontrolled trials were included as long as the study designs were randomized. Clinical as well as experimental studies that assessed the effects of analgesics after ovariohysterectomy in dogs were included. Only complete papers were included for review. Where only abstracts were available, full papers were obtained directly from the corresponding authors through the availed e-mail contacts. The systematic procedure followed to include and exclude articles is illustrated in Figure-1.

### Data extraction and synthesis

The articles that met the inclusion criteria were read in full, and data were extracted systematically in a predefined, standardized manner. Extracted data included the author, year of publication, study design, objectives of the study, analgesic drugs, technique of drug administration (multimodal vs. single drug therapy, pre-emptive vs. post-operative administration, course of drug administration, and epidural vs. systemic administration), and need for rescue analgesia. Quantitative data synthesis was carried out on homogeneous data. Homogeneity was achieved by grouping the data into categories based on several characteristics, which included overall goal of the study (analgesic efficacy, comparison of analgesia, timing effect, route effect, and dose effect), total number of dogs used, number of dogs per group, type of analgesic groups/therapies (nonsteroidal anti-inflammatory drugs [NSAIDs], opioids, NSAIDs-opioids, local analgesics, and acupuncture), analgesic protocols (individual or multimodal), timing of analgesic administration (pre-emptive, post-operative, or pre-emptive plus post-operative), course of analgesic therapy (once, 24 h, 48 h, and 72 h). The number of dogs that required rescue analgesia in each study (where available) was recorded, and further comparisons were carried out between different homogenous categories described above. The aim of these comparisons was to demonstrate the relative analgesic strength between the different types of drugs and their techniques of administration. Evaluation of an article for inclusion in the study as well as data extraction was performed independently by two reviewers. Any arising disagreements between the two reviewers were resolved through a discussion leading to a consensus.

## Results

### General demographics

#### Number of studies

**Figure-2:**
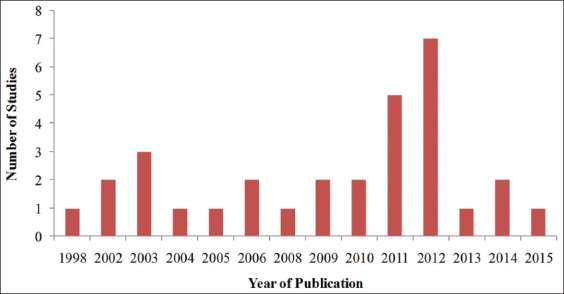
Distribution of studies that met the inclusion criteria of this systematic review based on their year of publication.

A total of 31 studies met the inclusion criteria for the systematic review (Table-1) [6-36]. The year with the highest number of studies that met the inclusion criteria was 2012 (22.6%), followed by 2011 (16.1%) and 2003 (9.7%). The distribution of studies as per their year of publication is illustrated in Figure-2.

**Table-1:** A summary of the studies that met the inclusion criteria, their objectives, and outcome.

Authors	Year of publication	Objective	Pain therapies	Dosage	Time of dosing	Course of admin	Rescue analgesia	Outcome
Ashraf and Abu-Seida [6]	2012	Evaluate efficacy	Diclofenac	1.1 mg/kg	Postop.	Once	No	Combination had similar analgesia to diclofenac alone but better analgesia compared to cefotaxime
			Cefotaxime	10 mg/kg				
			Diclofenac+Cefotaxime	1.1 and 10 mg/kg				
Buhari *et al*. [7]	2012	Evaluate efficacy	Tramadol IV	3 mg/kg	Preop.	Once	No	In IV analgesia is faster but of similar efficacy compared to SQ
			Tramadol SQ	3 mg/kg				
Camargo *et al*. [8]	2011	Compare analgesia	Firocoxib	5 mg/kg	Preop.	Once	Yes	Firocoxib has superior analgesia than butorphanol
			Butorphanol	0.2 mg/kg				
Campagnol *et al*. [9]	2012	Compare analgesia	Incisional bupivacaine	1 mg/kg	Preop.	Once	Yes	Intraperitoneal bupivacaine more effective than incisional bupivacaine
			Intraperitoneal bupivacaine	5 mg/kg				
Carpenter *et al*. [10]	2004	Compare analgesia	Bupivacaine	4.4 mg/kg	Postop.	Once	Yes	Intraperitoneal/incisional bupivacaine provides better analgesia than intraperitoneal/incisional lidocaine
			Lidocaine	8.8 mg/kg				
Cassu *et al*. [11]	2012	Compare analgesia	Electroanalgesia of acupoint EA		Preop.	Once	Yes	Acupoint EA and combination have better analgesia than dermatome
			Pre-incisional dermatome					
			Their combination					
Caulkett *et al*. [12]	2003	Compare analgesia	Meloxicam	0.2 mg/kg	Preop.	Once	Yes	Meloxicam has better analgesia than butorphanol
			Butorphanol	0.2 mg/kg				
Dzikiti *et al*. [13]	2006	Compare analgesia	Morphine	0.4 mg/kg	Preop.	24 h	Yes	Morphine, carprofen, and morphine-carprofen combination have similar analgesia
			Carprofen	4 mg/kg				
			Morphine-carprofen	0.4 and 4 mg/kg				
Frazílio *et al*. [14]	2014	Compare analgesia	Nalbuphine	0.3 mg/kg	Preop.	Once	Yes	Nalbuphine at 0.6 mg/kg provides superior and longer analgesia than at 0.3 mg/kg
			Nalbuphine	0.6 mg/kg				
Imagawa *et al*. [15]	2011	Compare analgesia	Dipyrone	Varying dosage 15, 25, and 35 mg/kg	Postop	48 h	Yes	Dipyrone 25 mg/kg and 35 mg/kg have similar analgesic efficacy better than achieved at 15 mg/kg
Kongara *et al*. [16]	2012	Compare analgesia	Morphine	0.5 mg/kg	Preop. and Postop.	Once	Yes	Analgesia produced by individual drugs is similar, but the combination provides superior analgesia
			Tramadol	3 mg/kg				
			Morphine-tramadol	0.1 and 3 mg/kg				
Lascelles *et al*. [17]	1998	Compare analgesia	Pre-operative carprofen	4 mg/kg	Preop. and Postop.	Once	No	Pre-operative carprofen has better analgesia than post-operative carprofen
			Post-operative carprofen	4 mg/kg				
Leece *et al*. [18]	2005	Compare analgesia	Carprofen	4 mg/kg SQ then 2 mg/kg oral	Preop. and Postop.	72 h	Yes	Both drugs have satisfactory analgesia, but meloxicam provides analgesia of longer duration than carprofen
			Meloxicam					
				0.2 mg/kg SQ then 0.1 mg/kg oral				
Lemke *et al*. [19]	2002	Evaluate efficacy	Ketoprofen	2 mg/kg	Preop.	Once	Yes	Ketoprofen reduces pain postoperatively
Mastrocinque and Fantoni [20]	2003	Compare analgesia	Morphine	0.2 mg/kg	Preop.	Once	Yes	Morphine and tramadol provide similar analgesia
			Tramadol	2 mg/kg				
Nunamaker *et al*. [21]	2014	Compare analgesia	Buprenorphine single release	0.2 mg/kg	Postop.	72 h	Yes	Both dosages provide similar analgesia with comparable side effects
				0.02 mg/kg				
			Buprenorphine					
Neves *et al*. [22]	2012	Compare analgesia	Tramadol	2 mg/kg	Preop.	Once	Yes	Extradural tramadol and morphine provide similar analgesia
			Morphine	0.1 mg/kg				
Pekcan and Koc [23]	2010	Compare analgesia	Transdermal fentanyl patch	50 or 75 ug/h	Preop.	Once	Yes	Epidural morphine provides better analgesia than transdermal fentanyl
				0.1 mg/kg				
			Epidural morphine					
Roija *et al*. [24]	2012	Evaluate efficacy	Magnesium sulfate	50 mg/kg	Preop.	Once	Yes	Magnesium sulfate failed to show any significant analgesic effects
Saritas *et al*. [25]	2015	Evaluate efficacy	Dexketoprofen	1.0 mg/kg	Preop.	Once	No	Dexketoprofen provides adequate analgesia
Selmi *et al*. [26]	2009	Compare analgesia	Vedaprofen	0.5 mg/kg	Preop.	Once	Yes	Vedaprofen provides similar analgesia to carprofen and ketoprofen
			Ketoprofen	2.2 mg/kg				
			Carprofen	2.2 mg/kg				
Shafford *et al*. [27]	2002	Compare analgesia	PEMF	0.5 HZ q 20 min	Postop.	Once	Yes	PEMF therapy provides adequate analgesia as does morphine and the combination
			Morphine	0.25 mg/kg				
			PEMF+morphine	0.5 HZ q 20 min and 0.25 mg/kg				
Shih *et al*. [28]	2008	Compare analgesia	Buprenorphine	0.02 mg/kg	Preop.	Once	Yes	Carprofen and the combination provide superior analgesia to that of buprenorphine; the combination has no added benefit
			Carprofen	4 mg/kg				
			Buprenorphine+carprofen	0.02 and 4 mg/kg				
Singh *et al*. [29]	2003	Compare analgesia	Pre-operative pentazocine	2 mg/kg	Preop. and Postop.	24 h	No	Pentazocine administered preoperatively has better analgesia than when given postoperatively
				2 mg/kg				
			Post-operative pentazocine					
Slingsby *et al*. [30]	2006	Compare analgesia	Varying dosages of sufentanil	10, 15, 25 µg/kg	Preop.	Once	Yes	Sufentanil provides better analgesia compared to carprofen; furthermore, it produces low pain score with increasing dosage
				4 mg/kg				
			Carprofen					
Slingsby *et al*. [31]	2011	Compare analgesia	Buprenorphine	20 µg/kg	Preop.	Once	Yes	Both dosages provide adequate analgesia but no significant advantage on higher dosages
			Buprenorphine	40 µg/kg				
Stanescu *et al*. [32]	2011	Compare analgesia	Robenacoxib	2 mg/kg	Postop.	72 h	Yes	Tramadol provides longer analgesia than robenacoxib
			Tramadol	2 mg/kg				
Tavakoli *et al*. [33]	2009	Evaluate efficacy	Metoclopramide	0.5 mg/kg	Preop.	Once	No	Metoclopramide is effective in reducing post-operative pain
Thengchaisri *et al*. [34]	2010	Compare analgesia	Carprofen	4.4 mg/kg	Postop.	72 h	No	Carprofen and tepoxalin provide better analgesia compared to vedaprofen
			Vedaprofen	0.5 mg/kg				
			Tepoxalin	20 mg/kg				
Tsai *et al*. [35]	2013	Compare analgesia	Meloxicam	0.2 mg/kg	Preop.	Once	Yes	Lidocaine provides comparable analgesia with meloxicam; the combination has no additive advantage
			Lidocaine	1.0 IV bolus then 0.025 CRI				
			Meloxicam-lidocaine	0.2-1.0 iv bolus then 0.025 CRI				
Vettorato and Bacco [36]	2011	Compare analgesia	Pethidine	5 mg/kg	Preop.	Once	Yes	Pethidine and butorphanol provide similar analgesia
			Butorphanol	0.4 mg/kg				

PEMF=Pulse electromagnetic field, Preop=Pre-operative, Postop=Post-operative, Admin=Administration, CRI=Constant rate infusion, IV=Intravenous, SQ=Subcutaneous, EA=Symbol of a specific acupoint in dogs

### Overall goals of the studies

Studies were carried out to compare the various effects of analgesics after ovariohysterectomy. A total of 58.1% (n=18) of the studies compared analgesia between different pain medications, 16.2% (n=5) evaluated efficacies of different drugs, 12.9% (n=4) compared the effects of various doses, 9.7% (n=3) compared the effects of route of drug administration, and 3.2% (n=1) compared the effects of timing (pre-operative or post-operative) of drug administration.

### Total number of dogs used in the studies

A total of 888 dogs were used in all the 31 studies that met the inclusion criteria. The mean number of dogs that were used per the study was 28.7±14.7 with the smallest number of dogs per the study being 12 and the highest number being 80. The mean number of dogs per group was 10.8±4.3 with the smallest number per group being 4 and the highest number being 20 dogs.

### Pain management practices

#### Analgesia protocols

Individual analgesic protocols were used in 83.9% (n=26) of these studies for managing pain in dogs after ovariohysterectomy, compared to 16.1% (n=5) of the studies that utilized multimodal drug therapy.

### Categories of analgesic drugs and techniques

Of the 31 studies that met the inclusion criteria, opioids were used in 38.7% of the studies, NSAIDs in 19.4%, the combination of NSAIDs and opioids in 19.4%, and local analgesia in 6.5% of the studies. The remaining therapies were used in equal measure of 3.2% of the studies as shown in Table-2.

**Table-2:** Categories of analgesic drugs and techniques used in dogs.

Category of analgesic	Number of studies	Percentage of the number of studies
Opioid	12	38.7
NSAID	6	19.4
NSAID and opioid	6	19.4
Local analgesic	2	6.5
Acupuncture	1	3.2
Acupuncture and opioid	1	3.2
Antiemetic	1	3.2
NMDA antagonist	1	3.2
NSAID and local analgesic	1	3.2
Total	31	100

NSAIDs=Nonsteroidal anti-inflammatory drugs, NMDA=N-methyl-D-aspartate

### Timing of analgesic administration

**Figure-3:**
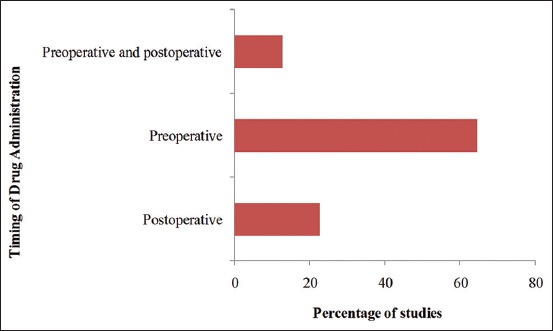
Timing of analgesic drug administration in dogs undergoing ovariohysterectomy.

The most preferred time for the administration of analgesics was before surgery (pre-operative), which was practiced in 64.5% (n=20) of the studies, followed by post-operative analgesia in 22.6% (n=7). In 12.9% (n=4) of the studies, analgesics were administered first preoperatively and then postoperatively (Figure-3). Furthermore, NSAIDs were administered mainly in the post-operative period (50%), while opioids (75%) and the NSAIDs-opioid drug combinations (66.7%) were mainly administered before surgery (preoperatively) as shown in Table-3.

**Table-3:** Timing for analgesic drug administration according to their categories in dogs undergoing ovariohysterectomy.

Timing of administration	Category of analgesics and percentage of studies in which they were used		

	NSAID	NSAID and opioid	Opioid
Post-operative	50.0	16.7	8.3
Pre-operative	33.3	66.7	75.0
Pre-operative and post-operative	16.7	16.7	16.7
Total	100.0	100.0	100.0

NSAIDs=Nonsteroidal anti-inflammatory drugs

### Post-operative course of analgesic administration

**Figure-4:**
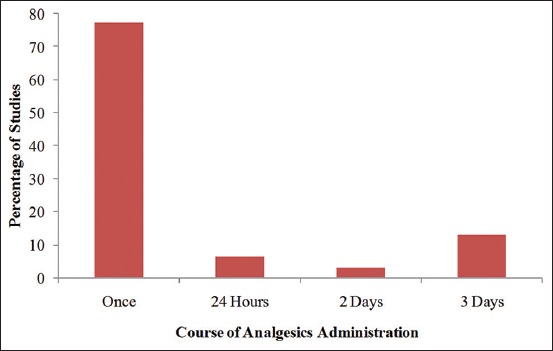
Course of analgesic administration for pain management after ovariohysterectomy in dogs.

Administration of analgesics only once postoperatively was the most common practice as reported in 77.4% (n=24) of the studies, while a 72-h post-operative course of analgesics was reported in 12.9% (n=4) of the studies. 24 h and 48-h courses of post-operative analgesic administration were reported in 6.5% (n=2) and 3.2% (n=1) of the studies, respectively, as shown in Figure-4.

### Requirement for rescue analgesia

Not all the studies assessed the need for rescue analgesia. However, a total of 713 dogs were used in the studies that assessed this parameter. Rescue analgesia was required in 25.5% (n=182) of these dogs.

### Comparison of the adequacy of analgesia between the drug protocols

More dogs in control groups required rescue analgesia postoperatively (57.3%) compared to dogs under pain therapy. The likelihood that a dog under single analgesic drug therapy (21.6%) would require rescue analgesia was twice as high as for a dog under multimodal analgesic drug therapy (11.3%) as shown in Table-4.

**Table-4:** The need for rescue analgesia in dogs under control, individual, and multimodal therapies.

Category	Total number of dogs in each protocol	Dogs requiring rescue analgesia in each protocol	Percentage of dogs requiring rescue analgesia in each protocol
Multimodal therapy	62	7	11.3
Individual drug therapy	555	120	21.6
Control group	96	55	57.3
Total	713	182	25.5

### Comparison between the categories of analgesics

Rescue analgesia was highest in dogs treated using acupuncture (43.8% of the dogs) and lowest in dogs treated using NSAID-opioids (8.6% of the dogs) (Table-5). Rescue analgesia was required in 9.3% of dogs treated using NSAIDs, 26.1% of dogs treated using opioids, and 28.6% of those under local analgesics.

**Table-5:** The requirement for rescue analgesia among the analgesic categories.

Analgesic category	Number of dogs requiring rescue analgesia/category	Total number of dogs/category	Percentage of dogs requiring rescue analgesia/category
NSAID-opioids	3	35	8.6
NSAIDs	15	162	9.3
Opioids	83	318	26.1
Local analgesic	14	49	28.6
Acupuncture	7	16	43.8

NSAIDs = Nonsteroidal anti-inflammatory drugs

### Comparison between the times of drug administration

**Figure-5:**
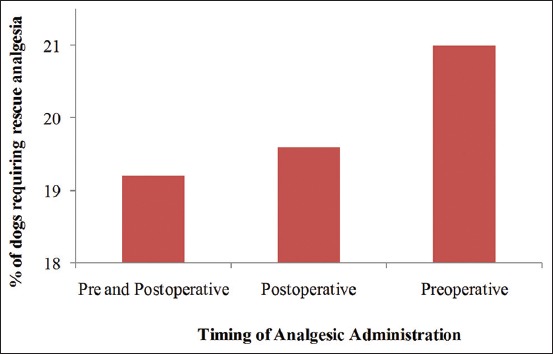
Requirement for rescue analgesia based on the timing of analgesic drug administration.

The number of dogs requiring rescue analgesia was lowest (19.2%) in categories of dogs that received pain medication both before and after surgery as compared to those that were given pain medication only before surgery (preoperatively) or only postoperatively. The highest number of dogs requiring rescue analgesia (21.0%) was witnessed in the category of dogs that were given analgesics only before surgery, followed by 19.6% of those dogs in the category that received analgesics only postoperatively (Figure-5).

### Comparison between the courses of drug administration

Only two groups (one-off and 72-h course of administration) were considered in the analysis of the requirement for rescue analgesia based on the course of drug administration. This was due to low numbers of studies in the other categories (24-h and 48-h courses of drug administration) and the fact that these two groups represented both extremes (short duration in one-off and long duration in 72-h). More dogs (26.4%) in the category that was given pain medication only once postoperatively required rescue analgesia as compared to 4.4% of dogs given analgesics over the course of a 72-h period (Table-6).

**Table-6:** Requirement for rescue analgesia between different courses of drug administration.

Course of administration	Number of dogs requiring rescue analgesia/category	Total number of dogs/category	Percentage of dogs requiring rescue analgesia
One-off	130	492	26.4
72 h	3	68	4.4
Total	133	560	23.8

## Discussion

This review indicates a general increasing trend in the number of studies focusing on post-operative pain after spaying in dogs, over a 20-year period. This observation suggests that veterinarians are becoming more aware of pain and its deleterious effects in surgical patients and are further exploring better therapies which can minimize pain and hence optimize surgical outcomes. This theory is further supported by the high number of studies that sought to gain a deeper understanding of comparative analgesic efficacy of the various drugs and techniques that can optimize analgesia provided by these agents. These techniques included individual drugs, drug combinations as well as varying the dosages, route, and timing of drug administration.

Opioids were the most commonly used analgesics followed by NSAIDs and NSAIDs-opioids drug combinations. Opioids are affordable and relatively available for use in developed countries where more than 90% of the reviewed studies were conducted, and this could explain, in part, their widespread use in managing pain in dogs as compared to NSAIDs and other analgesics. Further observations indicated that opioids were administered preoperatively and NSAIDs mostly postoperatively. Opioids are known to have good analgesia and sedative effects [1], and this could explain their widespread use for premedication. In addition, opioids are known to cause adverse effects postoperatively compared to NSAIDs, therefore limiting their use in post-operative period. The fact that NSAIDs have a longer duration of analgesia and lack sedative effects when compared to opioids [1] could have influenced their extensive use postoperatively where analgesia rather than sedation is required. However, since NSAIDs are known to minimize production of prostaglandins caused by trauma like in surgery [37], their use preoperatively would arguably result in better pain management as compared to when administered postoperatively. Although opioids are widely used for post-operative pain management in dogs, their administration is restricted to the period that the animal is hospitalized [38] due to their associated side effects. This limits their use in multimodal analgesia protocols in the home environment and may lead to inadequate analgesia and consequent “break-through” pain [38].

This study revealed that individual drug therapy was the more frequently used technique for pain management than multimodal therapy. This is probably a reflection of inadequate information available on the latter, particularly on the drugs that can be used together, the dosages, analgesic efficacy, and associated side effects. This limits the veterinarians to the use of individual drugs until sufficient reliable information on multimodal drug therapy is available to them.

Results, from this systematic review, show that NSAIDs had better pain relieving ability than opioids as indicated by the number of dogs requiring rescue analgesia. A similar observation has also been made in another systematic review conducted to assess the efficacy of NSAIDs and opioids in the treatment of acute renal colic in humans [39]. This is attributed to the fact that opioids act indirectly on the cause of pain through the opioids receptors [40], while NSAIDs act directly on prostaglandin release, which is the main intermediary of pain in surgery and most pathological processes in the body [37].

Local analgesics were also used in managing pain in dogs undergoing ovariohysterectomy and these included bupivacaine [9] and lidocaine [10,35]. Interestingly, the requirement for rescue analgesia in dogs given local analgesics was almost the same to that of dogs treated using opioids. This observation can be attributed to the fact that local analgesics were mostly administered directly at the site of nociceptor stimulation either at the skin incision (incisional) or at the ovarian stamps (intraperitoneal) [9,10] as compared to opioids that were administered systemically. Considering the cost, availability, restrictions, and the side effects associated with opioids compared to local analgesics, this observation is encouraging and could stir interest, leading to possible widespread use of this technique in dogs undergoing ovariohysterectomy.

Almost half of the dogs treated using acupuncture required rescue analgesia postoperatively. In addition, variations in the outcome of analgesia treatment were observed when different acupoints or acupuncture techniques were used. For example, a study by Cassau *et al*. [11] demonstrated that dogs treated by electrical stimulation of acupoint EA had lower pain scores compared to dogs treated at pre-incisional dermatomes. Based on these available studies, it can be inferred that the use of acupuncture for post-operative pain management, especially following ovariohysterectomy in dogs, produces variable outcomes and therefore is not as reliable as the proven therapies such as NSAIDs, opioids, and local analgesics.

More dogs in the control group required rescue analgesia more than those in which pain therapy was instituted. This observation confirms the fact that ovariohysterectomy in dogs is associated with post-operative pain, which has previously been described as acute and moderate [1]. For this reason, any dog undergoing this surgical procedure must receive pain medication at least for 24 h postoperatively to overcome deleterious physiological effects associated with pain and for humane reasons. Such deleterious effects include increased post-operative stress, immunosuppression, increased arterial blood pressure, delayed wound healing, negative protein balance, decreased food intake, and development of maladaptive behaviors such as self-mutilation [3,4]. The use of analgesics in pre-operative period in the form of opioids and alpha-2 adrenergic agonists could be the reason why only 57% and not all the dogs in the control groups required rescue analgesia.

Almost double the number of dogs that were treated using individual drug therapy required rescue analgesia compared to those treated using multimodal drug therapy. Studies have shown that irrespective of the dose used, a single class of analgesic drugs cannot provide complete analgesia due to the complex nature of pain transmission, which involves multiple pathways, mechanisms, and transmitter systems [41]. Multimodal drug therapy confers the advantages of using small doses of individual drugs but most importantly additive analgesia [19,42]. This improves the patient comfort and minimizes the need for high doses or prolonged use of any one particular drug [43], hence minimizing the likelihood of undesirable side effects. Furthermore, the widespread over-reliance on one class of drugs as is the case with NSAIDs is likely to not only undermanage some or perhaps many patients for their pain but also could increase the possibility of side effects associated with such drugs [43].

Administering drugs both before and after surgery was a technique associated with better outcomes compared to giving drugs either only before surgery or only after surgery. Several studies exist both in human and veterinary anesthesiology which demonstrate the beneficial effects of administering analgesics before surgery. For instance, carprofen administered preoperatively was shown to be more effective than one administered postoperatively in dogs undergoing ovariohysterectomy [17]. This beneficial effect can be attributed to: (1) Higher plasma levels of the drug at the time of surgery when given preoperatively; (2) higher levels of the drug in tissue fluid/inflammatory exudates when administered before surgery; (3) a positive pre-operative effect in terms of either decreasing the amount of noxious information generated at the periphery which decreases any central changes or blocking the entry of the noxious information into the spinal cord; and (4) high tissue levels of drug before the surgery which promotes a more effective action against local inflammation [17]. However, results from this study show that administering drugs postoperatively only had better outcomes compared to pre-operative administration. This is attributed to the fact that, since pain was assessed serially after surgery, the plasma concentration of drugs administered postoperatively was higher compared to the plasma concentration of those administered before surgery, resulting in the observed lower pain scores. Administering drugs both before and after surgery is then an innovative and effective way of managing pain as confirmed by the findings in this study. This technique utilizes the beneficial effects of each of the techniques (pre-operative and post-operative) resulting in better outcomes.

The need for rescue analgesia was very low in dogs that were given analgesics for 3 days compared to those that were given one-off pain medication, postoperatively. This observation might suggest that pain which occurs following ovariohysterectomy may be moderate but can last for several days. It could, therefore, make sense to administer analgesics more than once postoperatively, and if the dog is to be discharged immediately after recovery from anesthesia as is often common after ovariohysterectomy, then pain medications should be dispensed for the client to administer for a prescribed period and taken into account drug use regulatory environment in each jurisdiction.

## Conclusions

The following conclusions can be drawn from this study:

- Opioids are the mainstream analgesics that are used to manage pain in dogs undergoing ovariohysterectomy and that one-time drug administration, pre-operative, and individual drug therapy are the commonly used techniques.
- NSAIDs are more effective in managing post-operative pain in dogs undergoing ovariohysterectomy.
- Multimodal drug therapies, administration of analgesics before and after surgery, as well as a 72-h course of pain therapy are the practices that provide better outcomes in managing acute post-operative pain in dogs following ovariohysterectomy.
- Such analgesic drugs and techniques mentioned in these conclusions are recommended for optimal pain management following ovariohysterectomy as well as for good animal welfare practices.

## Author’s Contributions

WEM, EMM, JNM, PGM, and SWM were involved in the study design, data analysis, and writing of the manuscript. All authors read and approved the final manuscript.

## References

[1] Gayner J.S, Muir W.W, Gayner JS, Muir WW (2002). Acute pain management: A case-based approach. Handbook of Veterinary Pain Management.

[2] Hansen B.D (2005). Analgesia and sedation in the critically ill. J. Vet. Emerg. Crit. Care.

[3] Gwendolyn L.C, Carrol M.S (1996). How to manage perioperative pain. Vet. Med. J.

[4] Gaynor J.S (1999). Is postoperative pain management important in dogs and cats?. Vet. Med. J.

[5] Wagner A.E, Hellyer P.W (2002). Observations of private veterinary practices inColorado, with an emphasis on anesthesia. J. Vet. Med. Educ.

[6] Ashraf M.A, Abu-Seida A (2012). Efficacy of diclofenac sodium, either alone or together with cefotaxime sodium, for control of postoperative pain, in dogs undergoing ovariohysterectomy. Asian J. Anim. Vet. Adv.

[7] Buhari S, Hashim K, Goh Y.G, Mustapha N.M, Gan S.H (2012). Subcutaneous administration of tramadol after elective surgery is as effective as intravenous administration in relieving acute pain and inflammation in dogs. Sci. World J.

[8] Camargo J.B, Steagall P, Minto B.W, Lorena S.E.F, Mori E.S, Luna S.P.L. (2011). Post-operative analgesic effects of butorphanol or firocoxib administered to dogs undergoing elective ovariohysterectomy. Vet. Anaesth. Analg.

[9] Campagnol D, Teixeira-Neto F.J, Monteiro E.R, Restitutti F, Minto B.W (2012). Effect of intraperitoneal or incisional bupivacaine on pain and the analgesic requirement after ovariohysterectomy in dogs. Vet. Anaesth. Analg.

[10] Carpenter R.E, Wilson D.V, Evans A.T (2004). Evaluation of intraperitoneal and incisional lidocaine or bupivacaine for analgesia following ovariohysterectomy in dogs. Vet. Anaesth. Analg.

[11] Cassu R.N, Silva D.A, Filho T.G, Stevanin H (2012). Electroanalgesia for the postoperative control pain in dogs. Acta Cir. Bras.

[12] Caulkett N, Read M, Fowler D, Waldner C (2003). A comparison of the analgesic effects of butorphanol with those of meloxicam after elective ovariohysterectomy in dogs. Can. Vet. J.

[13] Dzikiti T.B, Joubert K.E, Venter L.J, Dzikiti L.N (2006). Comparison of morphine and carprofen administered alone or in combination for analgesia in dogs undergoing ovariohysterectomy. J. S. Afr.Vet. Assoc.

[14] Frazílio F.O, De Rossi R, Jardim P.H, Marques B.C, Martins A.R, Hermeto L.C (2014). Effects of epidural nalbuphine on intraoperative isoflurane and postoperative analgesic requirements in dogs. Acta Cir. Bras.

[15] Imagawa H.V, Fantoni D.T, Tatarunas A.C, Mastrocinque S, Almeida T.F, Ferreira F, Posso I.P (2011). The use of different doses of metamizole for post-operative analgesia in dogs. Vet. Anaest. Analg.

[16] Kongara K, Chambers J.P, Johnson C.B (2012). Effects of tramadol, morphine or their combination in dogs undergoing ovariohysterectomy on peri-operative electroencephalographic responses and post-operative pain. N. Z. Vet. J.

[17] Lascelles B.D, Cripps P.J, Jones A, Waterman-Pearson A.E (1998). Efficacy and kinetics of carprofen, administered preoperatively or postoperatively, for the prevention of pain in dogs undergoing ovariohysterectomy. Vet. Surg.

[18] Leece E.A, Brearley J.C, Harding E.F (2005). Comparison of carprofen and meloxicam for 72 hours following ovariohysterectomy in dogs. Vet. Anesth. Analg.

[19] Lemke K.A, Runyon C.L, Horney B.S (2002). Effects of preoperative administration of ketoprofen on anesthetic requirements and signs of postoperative pain in dogs undergoing elective ovariohysterectomy. J. Am. Vet. Med. Assoc.

[20] Mastrocinque S, Fantoni D.T (2003). A comparison of preoperative tramadol and morphine for the control of early postoperative pain in canine ovariohysterectomy. Vet. Anesth. Analg.

[21] Nunamaker E.A, Stolarik D.F, Ma J, Wilsey A.S, Jenkins G.J, Medina C.L (2014). Clinical efficacy of sustained-release buprenorphine with meloxicam for postoperative analgesia in beagle dogs undergoing ovariohysterectomy. J. Am. Assoc. Lab. Anim. Sci.

[22] Neves C.W, Balan J.A.O, Pereira D.R, Stevanin H, Cassu R.N (2012). A comparison of extradural tramadol and extradural morphine for postoperative analgesia in female dogs undergoing ovariohysterectomy. Acta Cir. Bras.

[23] Pekcan Z, Koc B (2010). The post-operative analgesic effects of epidurally administered morphine and transdermal fentanyl patch after ovariohysterectomy in dogs. Vet. Anesth. Analg.

[24] Roija E, Dzikiti B.T, Fosgate G, Goddard A, Stegmann F, Schoeman J.P (2012). Effects of a constant rate infusion of magnesium sulphate in healthy dogs anaesthetized with isoflurane and undergoing ovariohysterectomy. Vet. Anesth. Analg.

[25] Saritas Z.K, Saritas T.B, Pamuk K, Korkmaz M, Yaprakci M.V, Yilmaz O, Demirkan I (2015). Evaluation of preemptive dexketoprofen trometamol effect on blood chemistry, vital signs and postoperative pain in dogs undergoing ovariohysterectomy. Bratisl. Lek. Listy.

[26] Selmi A.L, Lins B.T, Cesar F.B, Figueiredo J.P, Duque J.C (2009). A comparison of the analgesic efficacy of vedaprofeno, carprofen or ketofen after ovariohysterectomy in bitches. Ciênc. Rural, Santa Maria.

[27] Shafford H.L, Hellyer P.W, Crump K.T, Wagner A.E, Ama K.R.M, Gaynor J.S (2002). Use of a pulsed electromagnetic field for treatment of post-operative pain in dogs: A pilot study. Vet. Anesth. Analg.

[28] Shih A.C, Robertson S, Isaza N, Pablo L, Davies W (2008). Comparison between analgesic effects of buprenorphine, carprofen, and buprenorphine with carprofen for canine ovariohysterectomy. Vet. Anesth. Analg.

[29] Singh G.J, Sangwan V, Gera S, Garg S.L (2003). Evaluation of pentazocine lactate as a postoperative analgesic in dogs. Haryana Vet.

[30] Slingsby L.S, Murison P.J, Goossens L, Engelen M, Waterman-Pearson A.E (2006). A comparison between pre-operative carprofen and a long-acting sufentanil formulation for analgesia after ovariohysterectomy in dogs. Vet. Anesth. Analg.

[31] Slingsby L.S, Taylor P.M, Murrell J.C (2011). A study to evaluate buprenorphine at 40 (µg/kg) compared to 20 (µg/kg) as a post-operative analgesic in the dog. Vet. Anesth. Analg.

[32] Stanescu M, Burac M.E, Diaconescu A.I, Togoe D, Vitalaru A, Birtoiu A.I (2011). Comparison of tramadol and robenacoxib postoperative analgesic efficacy in dogs. Scientific works. Series C. Vet. Med.

[33] Tavakoli A, Mehrjerdi H.K, Haghighi A (2009). Analgesic effects of metoclopramide following conventional ovariohysterectomy in bitches. Iran. J. Vet. Surg.

[34] Thengchaisri N, Sattasathuchana P, Niyom S, Chantornvong W (2010). Comparison of carprofen, vedaprofen and tepoxalin for postoperative analgesia and serum pge2 level in dogs after ovariohysterectomy. Thai J. Vet. Med.

[35] Tsai T.Y, Chang S.K, Chou P.Y, Yeh L.S (2013). Comparison of postoperative effects between lidocaine infusion, meloxicam, and their combination in dogs undergoing ovariohysterectomy. Vet. Anesth. Analg.

[36] Vettorato E, Bacco S (2011). A comparison of the sedative and analgesic properties of pethidine (meperidine) and butorphanol in dogs. J. Small Anim. Pract.

[37] Mathews K.A (1996). Nonsteroidal anti-inflammatory analgesics in pain management in dogs and cats. Can. Vet. J.

[38] Murrell J, Flaherty D (2014). Extending postoperative opioid analgesia in dogs 1. Oral drug administration. In Pract.

[39] Holdgate A, Pollock T (2004). Systematic review of the relative efficacy of non-steroidal anti-inflammatory drugs and opioids in the treatment of acute renal colic. Brit. Med. J.

[40] Reich J.D, Hanno P.M (1997). Factitious renal colic. Urology.

[41] Lascelles B.D.X (1999). Preoperative analgesia-opioids and NSAIDs. Waltham Foc.

[42] White P.F, Kehlet H, Neal J.M, Schricker T, Carr D.B, Carli F (2007). The role of the anaesthesiologist in fast-track surgery. From multimodal analgesia to perioperative medical care. Anaesth. Analg.

[43] Epstein M.E (2011). Transoperative Pain Management: A Framework.

